# Recognizing encephalopathy in immune checkpoint inhibitor therapy: A single‐center experience

**DOI:** 10.1002/cam4.3818

**Published:** 2021-03-03

**Authors:** Danmeng Wei, Daniel J. Zhou, Proleta Datta, Olga Taraschenko

**Affiliations:** ^1^ Department of Neurological Sciences University of Nebraska Medical Center Omaha NE USA

**Keywords:** altered mental status, encephalopathy, immune checkpoint blockade, immunotherapy for cancer, neurotoxicity

## Abstract

**Background:**

In this pilot study, we examined the characteristics of patients with and without central nervous system (CNS) malignancies who developed immune checkpoint inhibitor (ICI)‐induced encephalopathy.

**Methods:**

We identified adult patients treated with ICIs between 1 January 2013 and 9 May 2018 at our tertiary care center who developed encephalopathy within 30 days of the last dose of ICI without other explained causes. Demographic and clinical features were compared between patients with primary and metastatic malignant CNS tumors and those without.

**Results:**

Of the 480 patients treated with ICIs, 14 (2.9%) developed encephalopathy induced by nivolumab (8), pembrolizumab (4), and combined ipilimumab‐nivolumab (2). Median age was 64.5 years. Patients with CNS malignancies tolerated more treatment cycles and developed encephalopathy later than patients without CNS lesions (20 and 32 days, respectively, *p* = 0.04) following ICI initiation. Four of seven patients with CNS tumors developed new contrast‐enhancing lesions on brain imaging despite having no changes on imaging for a median of 61 (30–545) days. Electroencephalogram (EEG) revealed features of generalized dysfunction in patients in both cohorts. Two patients without and three with CNS malignancies were treated with steroids. Two thirds of patients without and 29% of those with CNS malignancies expired during ICI therapy or shortly thereafter.

**Conclusions:**

Lack of the uniform evaluation limits the definitive conclusion of the cause of encephalopathy in some patients but reflects the standard of care at the time of their assessment. ICI‐associated neurotoxicity presenting with encephalopathy is an ominous complication of ICI therapy, especially if left untreated. Prompt recognition and involvement of multidisciplinary care, including neurologists, would facilitate timely administration of recommended therapies.

## Graphical abstract

Patients receiving treatment with immune checkpoint inhibitors, a novel class of anticancer medications, can develop confusion, decreased responsiveness, headaches, and seizures that, if left untreated, can be fatal. We assessed the frequency of these neurological side effects in patients with and without tumors of the brain and compared their management at our institution. We found that the evaluation of patients in both groups was often incomplete. We also determined that the majority of patients were not treated according to the recommended guidelines and most of these patients were deceased shortly following their admission to the hospital. We concluded that the complications of immune checkpoint inhibitor therapy are not readily recognized by practitioners. We summarized the existing recommendations for the evaluation and treatment of these patients and proposed that neurologists be included in multidisciplinary efforts to treat these patients.

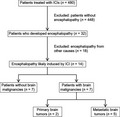

**Lay summary**

Patients receiving treatment with immune checkpoint inhibitors, a novel class of anticancer medications, can develop confusion, decreased responsiveness, headaches, and seizures that, if left untreated, can be fatal. We assessed the frequency of these neurological side effects in patients with and without tumors of the brain and compared their management at our institution. We found that the evaluation of patients in both groups was often incomplete. We also determined that the majority of patients were not treated according to the recommended guidelines and most of these patients were deceased shortly following their admission to the hospital. We concluded that the complication of immune checkpoint inhibitor therapy is not readily recognized by practitioners. We summarized the existing recommendations for the evaluation and treatment of these patients and proposed that neurologists be included in multidisciplinary efforts to treat these patients.

## 1 INTRODUCTION

Immune checkpoint inhibitors (ICIs), a novel class of antitumor monoclonal antibodies, have revolutionized treatment of relapsing advanced tumors of the skin, solid organs, and lymphatic system.^1^ These highly effective therapies interfere with the inhibitory effects of cytotoxic T‐lymphocyte antigen‐4 (CTLA‐4) and programmed cell death/programmed death ligand‐1 (PD1/PDL1) proteins in T‐cell regulatory pathways, resulting in markedly stronger activity of T cells against tumor cells.^1^

The immune activation may lead to ICI‐related neurological adverse effects (irAE‐N), manifested as weakness, fever, headache, altered mental status, abnormal movements, or seizures.^2^, ^3^ High‐grade central nervous system (CNS) toxicities were reported in fewer than 1% of patients treated with ICIs and developed with a median latency of 6 weeks.^4^, ^5^ The phenomena of pseudoprogression (increase in size due to therapy) and hyperprogression (doubling in size) of brain tumors following ICI therapy have also been reported.^6^, ^7^ However, only one patient was reported to present with encephalopathy.^8^

Evaluation of patients with suspected ICI‐related neurotoxicity includes cerebrospinal fluid (CSF) analysis, brain imaging, and EEG to assess for the presence of central inflammation, neuronal autoantibodies, and non‐convulsive seizures.^2^ If irAE‐N is confirmed, treatment involves discontinuing ICI and initiation of immunotherapy with steroids, intravenous immunoglobulins (IVIG), plasma exchange, rituximab, or natalizumab.^5^ However, the clinical management of encephalopathy in patients with primary or metastatic brain malignancies may require a different approach from that in patients without brain lesions. Specifically, the lumbar puncture recommended for the evaluation of encephalopathy related to the ICI therapy is contraindicated in most patients with space‐occupying CNS lesions due to the risk of herniation and thus it may not be performed.^9^ This is particularly applicable to patients who present with an altered mental status when the existence of an increased intracranial pressure cannot be reasonably ruled out. Therefore, the diagnosis of irAE‐N in these patients may need to be established without the CSF examination. Furthermore, some patients with CNS malignancies who are already receiving the corticosteroids to reduce cerebral edema may be managed differently than those without steroid use. The existing guidelines for the management of the irAE‐N related to the ICI therapy do not provide the distinctions specific to patients with CNS malignancies and the existing literature is limited.^10^, ^11^ Therefore, a consensus on specific approaches to the diagnosis and management of patients with and without brain malignancies is urgently needed.

Early diagnosis of irAE‐N in patients receiving ICIs may improve the prognosis.^3^ In this single‐center retrospective pilot study, we assessed the clinical course, management approaches, and outcomes of patients with ICI‐induced encephalopathy, with a focus on comparisons between groups with and without CNS malignancies.

## 2 METHODS

### 2.1 Study population

Retrospective chart review and analysis were conducted with the approval of the Institutional Review Board at the University of Nebraska Medical Center. Using a custom SQL query, we identified patients who were treated with ICIs from 1 January 2013 to 9 May 2018 and developed encephalopathy. The following key words were applied: “nivolumab”, “pembrolizumab”, “ipilimumab”, “atezolizumab”, “durvalumab,”, “avelumab”, “encephalopathy”, “confusion”, “altered mental status”, and “delirium”. Following the identification of these patients from the electronic medical record query, the clinical documentation was reviewed to extract the clinical, laboratory, radiological, and EEG data. A comprehensive metabolic panel, including liver function tests and complete blood count as well as brain images, needed to be documented for the patients to be included. Additional tests (i.e., blood cultures, CSF examination, and EEG) were performed at the discretion of the treating team.

### 2.2 Case identification

ICI‐induced encephalopathy was defined as a change from baseline cognitive status within 30 days of the last dose of ICI without other identified causes of encephalopathy. The inclusion criteria were as follows: being treated with the first or repeated cycles of ICIs for the systemic or CNS malignancy, being within 30 days from the last dose of the ICI, and being diagnosed with altered mental status, encephalopathy, or delirium upon presentation to the hospital. Patients with sepsis, clinically significant abnormalities in the sodium or calcium metabolism at the discretion of the treating team, acute kidney, respiratory, and hepatic failure, or those with underlying dementia were excluded.

Two neurologists with expertise in general and autoimmune epilepsy (P.D. and O.T.) reviewed all cases to ascertain final diagnoses. The severity of encephalopathy was established according to the classification outlined in the Common Terminology Criteria for Adverse Events (CTCAE), Version 5.^12^ We abstracted and compared demographic and clinical characteristics of patients with and without primary or metastatic CNS malignancies. Mann‐Whitney U tests and Fisher's exact tests were applied to compare continuous and categorical variables, respectively (GraphPad Software, Inc., San Diego, CA).

## 3 RESULTS

**FIGURE 1:**
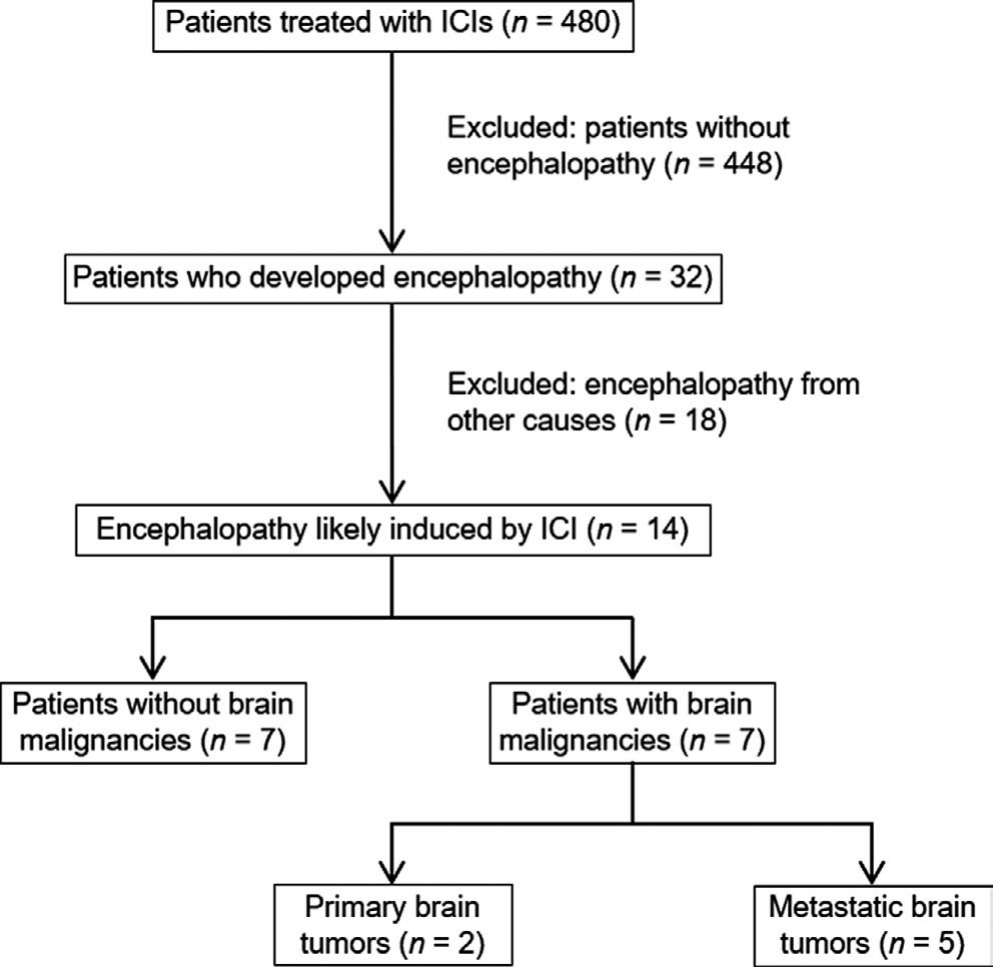
Flow chart of patient selection for the case series. ICI, immune checkpoint inhibitor

There were 480 adult patients who were treated with at least one ICI, such as atezolizumab (33), avelumab (4), durvalumab (20), ipilimumab (65), nivolumab (209), and pembrolizumab (149). Thirty‐two patients developed encephalopathy. Eighteen patients who developed encephalopathy due to other etiologies (e.g., abnormalities of sodium and calcium metabolism, urinary tract infection, pneumonia, sepsis, etc) were excluded (Figure 1). Of the remaining 14 cases, seven had primary (2) or metastatic tumors (5), and the other seven were without any malignant CNS tumors. Ten patients (2%) developed mild encephalopathy while four patients (0.8%) had severe encephalopathy. Lack of the EEG and CSF examination data precludes the definitive conclusion on the etiology of encephalopathy in some patients.

The patients without CNS malignancies had a median age of 63 (45–82) years, and 43% were male (Table 1, Supplemental Table S1). The median number of cycles of ICI therapy was one (1–3). The most common presenting neurologic features were acute confusion and generalized weakness, which developed with a median latency of 20 (3–50) days from the first ICI treatment (Tables 1 and 2). There were five and two patients with grades 2 and 3 encephalopathy, respectively. CSF was obtained in one patient. The fluid was unremarkable, although autoimmune antibody studies were not ordered. EEGs were obtained in four patients and demonstrated generalized slowing, background disorganization, and generalized periodic discharges (GPDs) with triphasic morphology (Table 2). Two patients received steroids and one had resolution of encephalopathy. Two other patients received supportive care and had transient improvement of mental status. The ICIs were held in five patients, while the treatment plan was not specified for two other patients who did not survive until their next treatment cycle. One patient died within 3 days of admission, and four died later by other causes (Table 2).

**TABLE 1:** Demographic and clinical characteristics of patients with ICI‐induced encephalopathy

	Without brain tumor (n = 7)	With brain tumor (n = 7)	*p*‐value
Chemotherapy regimen (number of patients)	N (2), P (3), I + N (2)	N (6), P (1)	
Median age, years (range)	63 (45–82)	65 (49–73)	0.97^a^
Sex (%)	F (57%), M (43%)	F (14%), M (86%)	0.27^b^
Median number of chemotherapy cycles prior to encephalopathy (range)	1 (1–3)	3 (1–10)	0.04^a^
Median latency to encephalopathy from first ICI cycle, days (range)	20 (3–50)	32 (9–133)	0.04^a^
Neurology consulted, % total	57%	57%	<0.99^b^
Treated with steroids, % total	29%	43%	<0.99^b^
Mortality at discharge, % total	14%	29%	<0.99^b^
Mortality by 30 days post‐discharge, % total	67%^c^	29%	0.29^b^

Abbreviations: F, female; I, ipilimumab; M, male; N, nivolumab; P, pembrolizumab.

^a^ Mann‐Whitney U test;

^b^ Fisher's exact test;

^c^ One value missing, unaccounted for in calculation.

**TABLE 2:** Clinical course and outcomes of patients with ICI‐induced encephalopathy

ID	Age (years) and sex	ICI, latency (days)	# of ICI cycles	Cancer type	EEG findings	AMS Grade	Initial brain imaging for AMS	Management	Outcomes
Patients without brain lesions									
1	82 F	I, N, 50	3	Metastatic neuroendocrine tumor	None	2	CT: Unremarkable	No steroids. Patient expired before next ICI dose	Expired in 3 days from onset of encephalopathy
2	78 M	P, 20	1	Metastatic neuroendocrine tumor	None	2	CT: Mild atrophy, white matter disease	No steroids. Patient discharged to comfort measures before next ICI.	Discharged on comfort measures, expired ≤30 days post‐discharge
3	81 F	N, 6	1	Non‐small cell lung cancer	None	2	MRI: Moderate volume loss, small vessel disease, small lacunar infarcts	No steroids, held ICI	Improved, ICI restarted, developed recurrent encephalopathy, discharged on comfort measures, expired ≤30 days post‐discharge
4^a^	45 M	I, N, 3	2	Neuroendocrine tumor	Background slowing and disorganization	2	MRI: Subependymoma, unchanged	Neurologist involved. Started oral prednisone, discontinued ICI	Mental status improved remarkably. Expired >30 days post‐discharge from other complications
5	48 F	N, 25	1	Non‐small cell lung cancer	Generalized slowing	3	MRI: Punctate subcortical foci, likely microangiopathy	Neurologist involved. No steroids, discontinued ICI	Discharged on comfort measures, expired ≤30 days post‐discharge
6	63 F	P, 27	2	Diffuse large B‐cell lymphoma	Generalized slowing, GPDs with triphasic morphology	2	MRI: Unremarkable	Neurologist involved. Started IV methylprednisolone, discontinued ICI	Discharged on comfort measures, expired >30 days post‐discharge
7	58 M	P, 6	1	Non‐small cell lung cancer	Moderate diffuse slowing	3	MRI: Diffuse cerebral volume loss, mild ventriculomegaly	Neurologist involved. No steroids, discontinued ICI	Improved, discharged home. Readmitted for AMS and discharged to hospice. Date of death unknown
Patients with brain lesions									
1	68 M	N, 113	6	Small cell and non‐small carcinoma of lung, brain metastasis	None	2	MRI: Left frontal lobe mass and 4 ring‐enhancing lesions in the left hemisphere; new from previous MRI	Continued previous oral dexamethasone, discontinued ICI	Improved
2	73 M	N, 32	7	Non‐small cell carcinoma of lung, brain and bone metastases	None	2	MRI: Multifocal small enhancing brain nodules consistent with metastatic disease	No steroids, ICI was held	Improved, ICI was restarted
3	61 M	N, 133	10	Non‐small cell carcinoma of lung, brain and spinal metastases	None	2	MRI: Multifocal metastatic disease to the brain, leptomeningeal metastatic disease to the brain	No steroids, ICI was held	Improved, ICI was restarted
4	61 M	N, 26	2	Anaplastic astrocytoma	None	2	MRI: Increased size of mass in right frontotemporal lobes and basal ganglia, suspicious for progression of tumor; evolution of prior right MCA infarcts	Neurologist involved, Started IV dexamethasone, resumed ICI	Discharged to home, presented to office for follow‐up then referred for comfort care. Expired >30 days post‐discharge.
5	49 M	P, 9	1	Esophageal cancer, brain metastases	Mild diffuse slowing	2	CT: Irregular area of increased attenuation in the interhemispheric fissure corresponding to probable meningeal metastases on previous MRI	Neurologist involved. No steroids. Patient expired before next ICI dose.	Deceased in 7 days from onset of encephalopathy
6	65 M	N, 29	3	Non‐small cell carcinoma of lung, brain metastases	Generalized slowing, focal slowing in right centroparietal region	3	MRI: New definitive contrast‐enhancing lesion and questionable lesion suspicious for metastatic disease	Neurologist involved. No steroids. Patient expired before next ICI dose	Deceased in 9 days from onset of encephalopathy
7^b^	69 F	N, 54	4	Glioblastoma multiforme	Severe diffuse slowing, frequent GPDs with triphasic morphology and sharp‐and‐slow wave discharges	3	MRI: Expansible FLAIR hyperintensity surrounding atrium of right lateral ventricle with minimal enhancement concerning for progressive disease	Neurologist involved. Started IV dexamethasone, discontinued ICI	Improved

Abbreviations: AMS, altered mental status; CSF, cerebrospinal fluid; CT, computerized tomography; EEG, electroencephalogram; F, female; FLAIR, fluid attenuation inversion recovery; I, ipilimumab; ICI, immune checkpoint inhibitor; IV, intravenous; M, male; MRI, magnetic resonance imaging; N, nivolumab; P, pembrolizumab; WBC, white blood cell count.

^a^ CSF analysis: glucose 77 mg/dL, protein 36 mg/dL, WBC 1/cm^2^;

^b^ CSF analysis: glucose 139 mg/dL, protein 55 mg/dL, WBC 10/cm^2^, neutrophils 53%.

In the cohort with primary or metastatic brain tumors and meningeal carcinomatosis, the median age (*p* = 0.97) and gender distribution (*p* = 0.27) were similar to those without CNS malignancies (Table 1, Supplemental Table S2). Five of seven patients received the ICIs for the progression of their primary systemic tumors and none of them had encephalopathy at the start of treatment. There were five and two patients with grades 2 and 3 encephalopathy, respectively, at the time of their admission. Patients with brain tumors tolerated more treatment cycles and developed encephalopathy 12 days later than patients without CNS tumors (*p* = 0.04). EEG was obtained in three patients and revealed mild to severe generalized slowing, interictal epileptiform discharges, and GPDs with triphasic morphology (Table 2). Four (57%) patients demonstrated new or evolved brain lesions on MRI compared to imaging obtained in a median of 61 (30–545) days prior to onset of encephalopathy. All three patients who received immunotherapy and two who were managed supportively improved, while two others were deceased within 9 days of developing encephalopathy (Table 2). The ICIs were stopped in four patients and continued in one patient, while two other patients have expired before their next scheduled cycle (Table 2). The overall mortality at discharge (*p* < *0*.*99*) and 30 days post‐discharge (*p* = 0.29) were similar in both groups (Table 1). The increase in the lesion number or size compared to the most recent previous brain imaging scans obtained at different time intervals was noted in patients 1, 4, 6, and 7 (Table 2, Supplemental Data). Therefore, the diagnosis of ICI‐associated encephalopathy in these patients was established with less certainty than in patients who had no changes on the brain imaging.

Neurologists were involved in the care of four (57%) patients in each group. Specifically, of 14 patients, three were treated in 2015, four in 2016, four in 2017, and three in 2018 (Supplemental Tables S1 and S2). The neurology team was not involved in the management of patients treated in 2015. None of these patients underwent EEG and CSF examinations or received steroids. In 2016, three of four patients were co‐managed by a neurologist who recommended EEG and CSF examinations in all patients and steroid therapy in two patients. In 2017, the neurology team was similarly involved in the management of three of four patients with ICI‐induced encephalopathy. In that year, all three patients managed by a neurologist underwent EEG, two had the CSF examination and one was treated with steroids. In 2018, one of three patients was managed by a neurologist and underwent EEG but did not receive steroids.

## 4 DISCUSSION

In this retrospective case series, we summarized and compared the clinical courses and outcomes of encephalopathy associated with the ICI therapy in cancer patients with and without malignant brain lesions. The temporal course of encephalopathy in relation to the start of the ICI therapy, along with a retrospective analysis of available diagnostic features and exclusion of other potential etiologies of altered mental status supported the relationship between the ICI and the onset of encephalopathy (Supplemental Tables S1 and S2). We found that 2% and 0.8% of patients treated with ICIs at our center developed mild and severe encephalopathy, respectively, that could not be attributed to other etiologies. This incidence is comparable to that previously reported rates in studies where patients with primary and metastatic brain cancers were excluded.^3^, ^5^

Neurologists were consulted in fewer than 60% of the cases and only five (36%) patients in our case series received conventional treatment for ICI‐related encephalopathy. Among the five patients treated with steroids, four were co‐managed by neurologists. It is likely that, in the remaining patients, the treating physicians at the time were not familiar with the phenomena of irAE‐N. However, our study was not large enough to determine if the inclusion of a clinician with neurology expertise or the assessment being performed more recently would improve the success of managing these patients. Further studies designed to assess the benefits of multidisciplinary approach in treating the irAE‐N will aid in refining the management guidelines for these patients. One patient in our study with a lung carcinoma metastatic to the brain was already receiving a steroid therapy at the time of manifestation of encephalopathy. Depending on the timing of steroid administration, the former may restrict tumor‐specific immune response to checkpoint inhibition by impairing T‐lymphocyte activation^13^; these adaptations may potentially influence the risk of developing the neurological toxicity during the therapy with ICIs. While it was shown that concurrent use of steroids was associated with the decreased overall response rate and survival in patients receiving ICIs for lung cancer, it is not clear whether the risk of neurotoxicity was similarly affected in these patients.^14^

While headaches, encephalopathy, and immune‐mediated meningitis in previous studies developed in a median latency of 6 weeks, the latency to ICI‐related encephalopathy in our study was much shorter.^4^ Interestingly, patients without malignant CNS disease developed cognitive deterioration much earlier than those with stable brain tumors. Possible explanations can be that the patients without brain lesions had more advanced stages of primary malignancies. Future prospective studies would allow to determine whether the location, histologic type, and stage of tumor contribute to the development of iatrogenic encephalopathy in patients receiving ICIs.

Mortality in both groups was high, with 29% and 67% of patients with and without brain tumors expiring by 30 days after discharge. These rates were higher than the reported 19% mortality among 200 cases of ICI‐induced encephalitis.^2^ However, in our study, ancillary tests were largely limited to brain imaging, and it is unclear whether the encephalopathy in patients who did not receive lumbar puncture or EEG was compounded by the presence of meningoencephalitis or non‐convulsive seizures.

Fifty‐seven percent of patients with CNS lesions and encephalopathy had an increase in lesion number or tumor size, which were stable in previous brain imaging. Although we did not apply formal radiological criteria to establish pseudo‐ or hyperprogression of the CNS tumors, we recognize that they may be potential contributors to encephalopathy. Reports on encephalopathy and pseudo‐ or hyperprogression of metastatic CNS tumors in the setting of ICI therapy remain limited.^8^, ^15^ The tumor enlargement could potentially represent an enhanced inflammatory response from the direct invasion of the immune effector cells at the CNS tumor site contributing to tumor necrosis and edema.^16^ Indeed, encephalopathy and focal interictal activity associated with pseudoprogression of metastatic CNS melanoma in response to ipilimumab were histologically confirmed to be accompanied by immune‐mediated inflammation and edema with extensive tumor necrosis.^16^ In some patients, such a response was accompanied by prolonged stabilization of the CNS tumor. This brings an intriguing possibility that an initial increase in tumor size may correlate with the high success of ICI therapy in malignant CNS disease.^16^ Patients with enlarging CNS tumors in our study were able to tolerate longer therapies before manifesting encephalopathy. This may reflect the limited CNS accessibility of ICIs and longer times required to mount an inflammatory response to the brain compared to tissues outside of the brain–blood barrier. Our study, however, was limited by its retrospective nature and small sample size that preclude the conclusions on the ICI‐induced phenomena of pseudo‐ and hyperprogression. Future studies with additional imaging that assesses the perfusion in brain tissues (e.g., positron emission tomography or MRI perfusion) may allow to discern inflammatory responses from disease progression in these patients.^7^

The retrospective design of this pilot study precludes the definitive differentiation of the ICI‐induced encephalopathy in all patients. Given that spinal fluid was obtained only in a few patients, an infectious encephalitis causing altered mental status could have been missed. Furthermore, despite the stability of the CNS tumors prior to initiation of the ICI therapy, it is possible that in some patients the change in tumor size was due to the progression of disease rather than the effects of ICIs. This limitation may weaken the results of this comparative study. The lack of differences between other clinical parameters in patients with and without tumor could have not been detected because of the small sample size. Furthermore, the generalizability of these results to other hospital settings may be limited because of the small number of subjects and the study site being a tertiary care center. None of the patients included in the report had an autopsy which limits the determination of an actual cause of death. While this adds to the limitations to the study it further underscores the lack of recognition of potential complications of immunotherapy leading to rapid demise in patients with cancer.

Our findings indicate that, in patients with mental status changes during the ICI therapy at our center, prompt assessment for ICI‐related causes of encephalopathy was largely not completed and neurologists were rarely participating in the management of encephalopathy. Further, the majority of patients did not receive immunosuppressive therapy to reverse the symptoms of encephalopathy. This was particularly relevant to the management of patients 5 and more years ago when the awareness of ICI‐induced neurotoxicity was lacking. The evaluation and management decisions for these patients were made according to the existing standards of clinical care prior to the publication of the National Comprehensive Cancer Network consensus guidelines in 2019.^17^ For example, the relevant recommendations from the Society for Immunotherapy of Cancer (SITC) Toxicity Management Working Group released in 2017 did not include EEG in the required diagnostic steps in suspected ICI‐related neurotoxicity.^18^ Given that ICIs are frequently being incorporated into treatment protocols of cancer patients, providers need to be aware of the potential risk of encephalopathy and be familiar with the appropriate workup. For patients suspected of having CNS toxicity related to the ICI, a consultation with clinician with neurology expertise and knowledge of the field of ICIs, brain imaging as well as serum and CSF analysis for the presence of autoantibodies should be requested.^5^ EEG should be obtained to exclude seizure activity. If a serious CNS toxicity is confirmed, the offending agent must be discontinued and specific immunotherapies be administered, including steroids, IVIG, and rituximab.

## 5 ETHICS APPROVAL STATEMENT

Retrospective chart review and analysis were conducted with the approval of the Institutional Review Board at the University of Nebraska Medical Center.

## CONFLICT OF INTEREST

The authors have no relevant conflict of interest to disclose.

## AUTHOR CONTRIBUTIONS

Danmeng Wei designed the study, collected the data, and drafted the manuscript for intellectual content. Daniel Zhou collected the data, performed the statistical analysis, and revised manuscript for intellectual content. Proleta Datta analyzed the data and revised manuscript for intellectual content. Olga Taraschenko directed the acquisition of data, interpreted the data, and revised manuscript for intellectual content.

## Supporting information

Supplementary Material

## Data Availability

All available data are included in the manuscript.
