# Supplementary material for: Recognizing encephalopathy in immune checkpoint inhibitor therapy: A single‐center experience
Source: Cancer Med. 2021 Mar 3;10(9):2978–86. doi: 10.1002/cam4.3818 (PMC8085930; doi:10.1002/cam4.3818)
Supplement: Supplementary file 1 — Supplementary Material [file CAM4-10-2978-s001.docx]

**Supplemental Data**

**Supplemental Results**

The information on tumor size and location at the time of evaluation for acute encephalopathy was extracted from radiology reports for all patients with primary and metastatic brain lesions. The dynamics of tumor lesions in each case were examined in reference to the most recent previous brain imaging.

In patient 1 (Table 2), the MRI brain obtained during the evaluation of acute encephalopathy revealed multiple ring-enhancing lesions, including a 5.8 cm (in diameter) lesion in the left frontal lobe, 1.8 cm lesion in the left superior occipital lobe, 2 cm lesion in the left posterior temporal lobe, and 1.2 mm lesion in the right cerebellum. These lesions were not found in previous brain imaging obtained 2 years prior to the onset of encephalopathy.

In patient 2 (Table 2), the MRI brain obtained at the onset of encephalopathy revealed scattered very small enhancing lesions within the bilateral cerebral hemispheres, consistent with stable or decreased metastatic disease compared to the previous imaging obtained 2 months ago. In patient 3 (Table 2), the MRI brain obtained during hospitalization for altered mental status revealed a persistent small region of gyral signal abnormality (size unspecified) within the inferior right temporal lobe, which was stable compared to MRI from 14 months prior.

In patient 4 (Table 2), MRI brain obtained during admission for acute encephalopathy revealed an enhancing mass in the right frontotemporal lobe and basal ganglia measuring 5.8 x 5.2 x 5.7 cm with increased surrounding vasogenic edema. The size of the lesion was somewhat increased from the previous imaging scan obtained 1 month prior, when it was 5.4 x 4.7 x 4.7 cm.

In patient 5 (Table 2), CT head obtained at the onset of encephalopathy revealed a thin, irregularly shaped area of signal intensity along the anterior border of the falx which was unchanged from previous imaging obtained 1 month ago. In patient 6 (Table 2), MRI brain revealed a contrast enhancing lesion abutting the falx posteriorly adjacent to the right parietal lobe and measuring 1 cm in diameter. There was also a questionable 3 mm contrast-enhancing lesion in the right occipital lobe. The previous imaging was obtained 2 months prior. In patient 7 (Table 2), the MRI brain obtained during the evaluation of acute encephalopathy revealed a new area of increased signal intensity in multiple areas, most conspicuously surrounding the atrium of the right lateral ventricle and right parietal cortex, which was not present in brain imaging 2 months prior.

**Supplemental Table 1.**

| ID | Exam #1 | Exam #2 | Exam #3 | Start of the first ICI | Onset of encephalopathy |
| --- | --- | --- | --- | --- | --- |
| 1 | Liver mass biopsy, 7/3/2017: Metastatic small cell neuroendocrine carcinoma |  |  | 12/6/2017 | 1/25/2018 |
| 2 | Bladder biopsy, 8/22/2017: Small cell neuroendocrine carcinoma |  |  | 5/11/2018 | 5/11/2018 |
| 3 | Liver nodule biopsy, 1/8/2015: Non-small-cell carcinoma, favor poorly differentiated adenocarcinoma, morphocytologically consistent with a pulmonary non-small cell carcinoma primary |  |  | 6/24/2015 | 6/30/2015 |
| 4^a^ | Liver biopsy, 6/26/2017: Well-differentiated neuroendocrine tumor with an elevated proliferation rate (grade 3) | ^a^ CSF cytology, 11/29/2017: No malignant cells | Esophageal biopsy, 1/30/2018: Neuroendocrine carcinoma | 11/22/2017 | 11/25/2017 |
| 5 | Bronchoalveolar lavage, 10/5/2015: Malignant cell consistent with adenocarcinoma | Right thigh biopsy, 10/8/2015: Metastatic poorly differentiated adenocarcinoma |  | 4/29/16 | 5/24/2016 |
| 6 | Colon biopsy, 8/5/2008: Diffuse large B-cell lymphoma | Inguinal lymph node biopsy, 12/30/2015: Diffuse large B-cell lymphoma | Bone marrow aspirate, 4/7/2016: Hypercellular bone marrow with trilineage dysplasia | 1/14/16 | 2/10/2016 |
| 7 | Liver node biopsy, 5/2/2018: metastatic squamous cell carcinoma |  |  | 5/24/18 | 5/30/2018 |

**Supplemental Table 2.**

| ID | Exam #1 | Exam #2 | Exam #3 | Start of the first ICI | Onset of encephalopathy |
| --- | --- | --- | --- | --- | --- |
| 1 | Lung biopsy, 10/13/2014:  Invasive moderately differentiated squamous cell carcinoma | Lung resection, 12/12/2013:  Invasive squamous cell carcinoma with associated carcinoma in situ | Bran biopsy,  10/16/2015:  Metastatic small cell carcinoma | 6/9/2015 | 9/30/2015 |
| 2 | Lumbar spine biopsy,  8/21/2012:  Metastatic adenocarcinoma consistent with lung primary | Lung, wedge biopsy, 9/17/2012:  Metastatic adenocarcinoma, lymphangitic pattern |  | 11/20/2015 | 12/22/2015 |
| 3 | Conus/cauda equina biopsy, 8/7/2015:  Metastatic poorly differentiated adenocarcinoma with immunophenotype suggestive of lung origin |  |  | 10/7/2016 | 2/17/2017 |
| 4 | Right temporal lobe biopsy, 12/16/2014:  High grade astrocytoma |  |  | 6/9/2016 | 7/5/2016 |
| 5^a^ | Esophageal biopsy,  1/25/2016: Invasive moderately differentiated adenocarcinoma | Bone and muscle biopsy,  5/17/2016:  Metastatic adenocarcinoma | ^a^ CSF cytology, 2/10/2017:  Tumor cells with cytomorphologic similarity to the adenocarcinoma | 6/22/2017 | 7/1/2017 |
| 6 | Lung biopsy, 7/26/2013:  Non-small cell carcinoma, favor poorly differentiated adenocarcinoma |  |  | 4/21/2016 | 5/20/2016 |
| 7^b^ | Right parietal tumor biopsy, 3/8/2016:  Glioblastoma, WHO grade IV, with extensive necrosis | ^b^ CSF cytology,  3/15/2017: Rare atypical cells |  | 1/20/2017 | 3/15/2017 |

**Supplemental Legends**

**Supplemental Table 1.** Biopsy specimen sampling and cerebrospinal fluid (CSF) cytology reports of the patients with systemic malignancies and immune checkpoint inhibitor (ICI)-associated encephalopathy. ^a^ CSF, cerebrospinal fluid analysis: WBC, white blood cell count 1, glucose 77 mg/dL, protein 36 mg/dL.

**Supplemental Table 2.** Biopsy specimen sampling and CSF cytology reports of the patients with primary and metastatic brain tumors and ICI-associated encephalopathy. ^a^ CSF, cerebrospinal fluid analysis: WBC, white blood cell count 5, glucose 80 mg/dL, protein 61 mg/dL. ^b^ CSF analysis: WBC 10, lymphocytes 10%, neutrophils 53%, glucose 139 mg/dL, protein 55 mg/dL.
